# Common Themes in Zoonotic Spillover and Disease Emergence: Lessons Learned from Bat- and Rodent-Borne RNA Viruses

**DOI:** 10.3390/v13081509

**Published:** 2021-07-31

**Authors:** Evan P. Williams, Briana M. Spruill-Harrell, Mariah K. Taylor, Jasper Lee, Ashley V. Nywening, Zemin Yang, Jacob H. Nichols, Jeremy V. Camp, Robert D. Owen, Colleen B. Jonsson

**Affiliations:** 1 Department of Microbiology, Immunology and Biochemistry, The University of Tennessee Health Science Center, Memphis, TN 38163, USA; ewilli99@uthsc.edu (E.P.W.); bspruill@uthsc.edu (B.M.S.-H.); mtayl121@uthsc.edu (M.K.T.); jlee175@uthsc.edu (J.L.); jnicho60@uthsc.edu (J.H.N.); 2 Department of Clinical Pharmacy and Translational Sciences, The University of Tennessee Health Science Center, Memphis, TN 38163, USA; ashnywe@uthsc.edu; 3 Department of Cell and Molecular Biology, St. Jude’s Children’s Hospital, Memphis, TN 38105, USA; zemin.yang@stjude.org; 4 Center for Virology, Medical University of Vienna, 1090 Vienna, Austria; jeremy.camp@meduniwien.ac.at; 5 Centro para el Desarrollo de Investigaciones Científicas, Asunción C.P. 1371, Paraguay; rowen@pla.net.py; 6 Department of Biological Sciences, Texas Tech University, Lubbock, TX 79409, USA

**Keywords:** spillover, zoonosis, RNA viruses, bats, rodents, outbreak, reservoir

## Abstract

Rodents (order Rodentia), followed by bats (order Chiroptera), comprise the largest percentage of living mammals on earth. Thus, it is not surprising that these two orders account for many of the reservoirs of the zoonotic RNA viruses discovered to date. The spillover of these viruses from wildlife to human do not typically result in pandemics but rather geographically confined outbreaks of human infection and disease. While limited geographically, these viruses cause thousands of cases of human disease each year. In this review, we focus on three questions regarding zoonotic viruses that originate in bats and rodents. First, what biological strategies have evolved that allow RNA viruses to reside in bats and rodents? Second, what are the environmental and ecological causes that drive viral spillover? Third, how does virus spillover occur from bats and rodents to humans?

## 1. Introduction

Globalization, environmental and anthropogenic changes provide ample opportunities for spillover and emergence of zoonotic diseases [1,2,3]. The term zoonosis was first coined in the latter half of the 19th century by Rudolf Virchow, who noted the relationship of humans and animals in the occurrence of infectious diseases [4]. Today, we recognize that greater than half of all human infectious diseases are zoonotic, a majority of which originated through the cross-species transmission of RNA viruses from wildlife to humans [5,6,7,8]; and, at present, we know of more than 224 RNA viruses that cause human disease with 88% of these being zoonotic in nature [9,10]. Of those zoonotic viruses that have spilled over, only a few have successfully adapted to humans and resulted in pandemics in the past two centuries, such as the 1918 H1N1 influenza A virus, human immunodeficiency virus (HIV), hepatitis C virus (HCV), and severe acute respiratory syndrome-coronavirus-2 (SARS-CoV-2). The 1918 influenza pandemic, which reached a global death toll of up to 100 million people, began as a spillover of an avian influenza H1N1 virus from a bird or some other animal such as pigs [11]. In the case of HIV, which has resulted in approximately 32.7 million deaths from AIDS-related illnesses (through the end of 2019), the ancestral virus spilled over from chimpanzees to humans [12]. The original reservoir of HCV, a virus that infects over 70 million worldwide, is still unknown. Viruses belonging to the same *Hepacivirus* genus have been isolated from reservoirs such as dogs, rodents, and horses [13]. The coronavirus disease 2019 (COVID-19) pandemic caused by SARS-CoV-2 may have started with the spillover of the virus from a bat to an intermediate host [14,15]; but neither the reservoir nor any intermediate host has been confirmed yet.

Most viruses that spillover from wildlife to humans do not typically result in pandemics [7]. More commonly, following infection, humans are “dead-end” hosts, and the virus is not transmitted further from person to person. Typically, these outbreaks are limited to the geographic distribution of the reservoir, but collectively, they have resulted in hundreds of thousands of infections and case fatalities. Some of the more notable, and geographically bound, wildlife to human outbreaks involves viruses harbored by rodents such as hantaviruses (e.g., *Hantaan orthohantavirus* (HTNV) and *Sin Nombre orthohantavirus* (SNV)) and arenaviruses (e.g., *Machupo mammarenavirus* (MACV), *Lassa mammarenavirus* (LASV), and *Argentinian mammarenavirus* (formerly Junin virus (JUNV))) found circulating in the Americas or Africa (Supplementary Table S1). While bats have a much greater geographical range, human outbreaks of viruses carried by bats such as *Nipah henipavirus* (NiV) and *Hendra henipavirus* (HeV) are limited to the geographical range of the bat species (Supplementary Table S2). 

In this review, we focus on three questions regarding zoonotic viruses that originate in bats and rodents. First, we discuss biological strategies that have evolved that allow RNA viruses to reside in bats and rodents. Second, we look at some of the environmental and ecological causes that drive spillover. Third, we discuss how spillover occurs from bats and rodents to humans by highlighting some shared and unique characteristics of previous epizootic events.

## 2. What Biological Strategies Have Evolved That Allow RNA Viruses to Reside in Bats and Rodents?

The two most abundant and globally distributed mammalian groups are rodents (order Rodentia) followed by bats (order Chiroptera). According to the Mammal Diversity Database maintained by the American Society of Mammologists, there are 2590 extant species within the Rodentia and 1430 extant species within the Chiroptera. Together, these represent 63% of the 6410 mammalian species known to exist today. Orders Rodentia and Chiroptera diverged approximately 96 million years ago (MYA) according to the most recent available published data (65 studies ca. December 2020) in TimeTree [16,17]. Both mammalian orders have been cited as harboring proportionally high richness of pathogenic viruses [8], and this may be directly related to the high diversity in these two mammalian taxa. The two families known to harbor zoonotic RNA viruses within the order Rodentia are Muridae and the Cricetidae with 843 and 809 species, respectively [18]. Over 50% of rodent-borne zoonotic viruses are members of two families, *Hantaviridae* and *Arenaviridae* [19]. Chiroptera is the second most species-rich group of mammals with 21 families [20]. Hence, it is not surprising that over 10,000 RNA virus sequences have been identified in various bat species, several of which are known to cause human disease [21,22,23,24,25]. Viral sequences noted in bats include *Astroviridae*, *Coronaviridae*, *Circoviridae*, *Adenoviridae*, *Filoviridae*, *Parvoviridae*, *Poxviridae*, *Picornaviridae*, and *Rhabdoviridae* [24,26,27,28,29,30,31,32,33]. Of these, only a small percentage of viruses harbored by bats from the rhabdoviruses, filoviruses, paramyxoviruses, and coronaviruses have been associated with disease and outbreaks in human populations. In addition, although bats are reservoirs of several viruses that have been responsible for serious outbreaks, models have shown that viruses harbored by bats are no more likely to be zoonotic than viruses that are harbored or transmitted by rodents or other hosts [19]. For some viruses in the genus *Ebolavirus*, bats are the presumptive main reservoir, but have yet to be definitively confirmed [34,35,36,37]. 

In the following section, we discuss how biological factors and lifestyle traits contribute to the ability of bats and rodents to serve as a reservoir for an RNA virus. For bat and rodent reservoirs of RNA viruses, multiple characteristics may contribute to their ability to serve as a reservoir [38,39,40,41]. The transmission of bat-borne viruses to humans through an intermediary host further complicates the biology. Moreover, various combinations of strategies have evolved that are not universal across all virus-reservoir host relationships, which include but are not limited to, limiting the pathogenesis of the viral infection, the ability of the virus-reservoir interplay to modulate the immune response such that the virus may persist, the behavioral and demographic characteristics of bats and rodents that promote endemic maintenance of the viruses they harbor, and the inherent genetic plasticity of RNA viruses. There is a lack of literature for many viruses in conjunction with their natural reservoir host in each of these areas, so we present examples of key areas of research that would benefit from continued research in the natural reservoir host and in their intermediate spillover hosts.

### 2.1. Pathogenesis of the Viral Infection

Numerous studies have reported that with some exceptions (e.g., lyssaviruses in bats, mammarenaviruses in rodents), many RNA viruses may infect and persist in bats and rodents without causing disease [42,43,44,45,46]. The ability of a mammal to carry a virus while healthy would contribute to maintenance and spread of the virus in the population. However, as stated, there are also reports of host reservoirs of viruses that experience disease or a loss in fitness. For example, pathology of bats following experimental infection with rabies viruses in the genus, *Lyssavirus*, has been reported [47,48,49]. However, in one report, viral antigen was found in the brain with no apparent pathology [50]. In addition to rabies, pathology (morbidity and mortality) has been demonstrated following experimental infection of *Artibeus jamaicensis*, the Jamaican fruit-eating bat, with a high dose (not the low dose) of *Tacaribe mammarenavirus* (TCRV) [51]. TCRV has been isolated from 11 species of bats in Trinidad. However, whether Jamaican fruit-eating bat is the “real” reservoir of TCRV remains to be seen—in fact, fruit bats, in general, may not be reservoirs as persistent infection with shedding has yet to be demonstrated [52]. In studies of rodent reservoirs, there is evidence of some disease in the natural reservoir. Anemia, splenomegaly, and decreased survival have been observed in MACV-infected *Calomys callosus* (large vesper mouse) [53]. In addition, Webb et al. [53] found the female *C. callosus* infected with MACV produce only 5% of the expected number of viable offspring, although having normal estrus cycles. MACV does not cause illness or death in either adult or juvenile mice, and as with other arenaviruses, it infects a variety of cell types. Although infectious virus has been recovered from most major organs, the kidney and spleen are the important target organs for arenavirus replication in rodents [54,55]. For rodent reservoirs of hantaviruses, there are examples of a loss in adult survival of animals based on ecological studies of hantaviruses in nature; for example, SNV infection decreases survival of deer mice (*Peromyscus maniculatus*) in the USA [56] and in bank vole (*Myodes glareolus*) populations harboring *Puumala orthohantavirus* (PUUV) surveyed in 55 areas over three years in Finland [57].

Although it is clear that accepted, or presumed reservoir hosts, have an immunological response to virus infection, no apparent disease is detected in experimental or natural infections of certain hosts with viruses such as some coronaviruses, henipaviruses, some hantaviruses, paramyxoviruses, and filoviruses. In an interesting study by Watanabe et al. [58] laboratory Leschenault’s rousette bats (*Rousettus leschenaultii*) were experimentally infected with a wild-caught *Betacoronavirus* isolated from the lesser short-faced fruit bat *(Cynopterus brachyotis*) in the Philippines, but these animals showed no clinical signs; viral RNA was only detected in the intestines and not in the liver, kidney, lung, spleen or brain. Similarly, Munster et al. [59] experimentally infected laboratory Jamaican fruit-eating bats with middle east respiratory syndrome-coronavirus (MERS-CoV). Although they could detect shedding of virus, they also reported no clinical signs or pathology. These limited studies of coronaviruses may suggest that an acute infection with virus shedding, but no pathology, may be common in bats, regardless of whether the species is a reservoir host. In other laboratory studies, Egyptian rousette bats, *Rousettus aegyptiacus*, were infected with *Marburg marburgvirus* (MARV), and no deaths, overt signs of morbidity, or gross lesions were identified, although, microscopic pathological changes were seen in the liver of infected bats [60,61,62,63,64]. In experimental studies by Halpin et al. [65], wild-caught pteropid bats in Australia were infected with the henipaviruses HeV and NiV. Bats showed virus shedding, but histopathology showed essentially no convincing pathology and no detection of viral antigen. Similarly, Middleton et al. [66] infected grey-headed flying fox bats (*Pteropus poliocephalus*) with NiV and reported infection, and pathological examination of tissues did not confirm the presence of the virus with associated pathology so that lesions could not be attributed to viral infection. Woon et al. [67] also infected black flying foxes (*Pteropus alecto*) with HeV and showed virus replication in the lungs and an inflammatory response, but no viral shedding nor pathology. Experimental infection of Egyptian rousette bats with the paramyxovirus, *Sosuga pararubulavirus*, showed infection and virus shedding, but no morbidity nor mortality. However, they report subclinical disease in a subset of tissues [68].

Uniquely for bats, one proposed explanation for the absence of disease for some viral infections is the “flight as fever” hypothesis which posits that the high metabolic rate required for flight stimulates a fever response [39,69]. Daily metabolic surges during flight leads to DNA damage caused by the production of reactive oxygen species [70]. Evidence suggests that during evolution, bats positively selected for genes involved in DNA repair which could have influenced their antiviral immune responses [45,71]. Moreover, to avoid DNA-mediated immunopathology induced during flight, bats have an altered DNA sensing system which may limit viral replication [45,72]. One study suggests virus replication within bat cells does not seem to be temperature dependent [73], suggesting that other characteristics of bats may contribute to enabling them to serve as viral reservoirs. 

### 2.2. The Host Immune Response

The ability of bats and rodents to modulate the immune response to viral infection has been an active area of investigation and has been recently reviewed elsewhere [39,42,45,74,75]. We highlight a few examples of how viruses interact with specific components of the bat or rodent host immune system, which results in a low level of viral infection or an ability of the host to tolerate viral replication [42,45,74,75,76,77,78]. In general, immune responses of the host play a major role in controlling the viral infection and viruses can exploit these responses in at least one of three ways; (i) immune ignorance, (ii) immune escape, and (iii) immune dysfunction (or exhaustion). The first immune responses to an initial infection occur in three phases in this general order: (i) innate immunity; (ii) early induced immunity; and (iii) adaptive immunity. The goal of each of these responses is the removal of the infectious agent. The innate response plays an important role in detection of the virus during acute stages of infection, which may set the stage for the magnitude of the adaptive responses.

#### 2.2.1. Interferons (IFN) and Interferon Stimulated Genes (ISGs)

Most viruses are indirectly sensitive to the induction of IFNs following infection as they induce the upregulation of ISGs; the proteins transcribed from the ISGs actively engage in an antiviral response that directly or indirectly thwart virus replication. Thus, many RNA viruses have evolved strategies to suppress the IFN response and studies suggest similarity of the innate immune signaling pathways in bats and rodents [45,79], although some of their counterparts in bats [80,81] and rodents may function differently. While an active area of research effort, there are few studies of the innate immune response in the many reservoir hosts with the virus they harbor. 

Research on bat IFNs suggested diversity in the expression of IFN-alpha. In the black flying fox (reservoir of henipaviruses), and the lesser short-nosed fruit bat IFN-alpha is expressed constitutively [82]. This is not observed in the reservoir of MARV, the Egyptian rousette bat [44]. When compared to ancestral bat species, the IFN gene loci of the black flying fox have contracted whereas the loci of Egyptian rousette bats have diversified [44,82]. Banerjee et al. [80] show that IFN-beta was expressed in big brown bat, *Eptesicus fuscus*, cells stimulated with poly(I:C); however, tumor necrosis factor-alpha, TNF-alpha, was much lower, and suppressed. In one study with Egyptian rousette bats experimentally infected with MARV, Guito et al. [83] observed a moderate elevation of innate antiviral genes, but not cytokines and adaptive immunity-related genes. The authors suggest that Egyptian rousette bats may have evolved a mechanism to control virus replication without induction of the adaptive responses. 

Two studies have examined henipaviruses in an immortalized cell line derived from the black flying fox [84,85]. The first study suggested that neither HeV or NiV infection results in type I and III IFN induction [85]. Both viruses also inhibit interferon signaling. In the second study, the bat cells were treated with supernatant from CHO cells that contained black flying fox IFN-γ, and they showed inhibition of virus replication [84]. Examinations of the antiviral response in rodent primary cells using the zoonotic viruses are limited. Studies of hantaviruses and their reservoirs suggest that viral strategies to antagonize the IFN response may vary [86]. Permanent reservoir cell lines that have been developed for the common vole (*Microtus arvalis*) and bank vole for hantaviruses will provide a valuable tool to exploring virus-reservoir host interaction [87]. 

The Myxovirus resistance (Mx) proteins are ISGs that are well-studied in in-bred laboratory rodents and shown to directly inhibit many RNA viruses (reviewed in [88]). In wild caught bank voles from various endemic regions in Europe, Dubois et al. [89,90] showed that bank voles from PUUV endemic regions in Europe are more tolerant to infection (higher viral load and delayed antibody production) and have lower ISG and cytokine expression (Mx2, TNF-alpha) compared to bank voles from non-endemic regions. 

The Mx1 gene was cloned from three families of bats: Pteropodidae, Phyllostomidae, and Vespertilionidae [91]. Examination of the ability of each of the bat Mx proteins confirmed their ability to reduce replication of *Zaire ebolavirus* (ZEBOV), vesicular stomatitis virus and *Rift Valley fever phlebovirus* (RVFV) [91]. However, the Mx from common pipistrelle, *Pipistrellus pipistrellus* did not show in vitro antiviral activity against RVFV, suggesting functional differences may exist among bats. Direct examination of immune signaling in primary bat cells from a reservoir with the virus they harbor has not been reported for any of these viruses. 

#### 2.2.2. B and T-Cells of Bats and Rodents

Bats potentially have more VDJ germline gene segments when compared to other mammals and with this lies the potential for bats to have a much larger naive immunoglobulin repertoire. For example, the naive B cell repertoire of the little brown bat (*Myotis lucifugus*) may potentially be more than 70,000 specificities [92]. Having a much larger repertoire suggests that the bat has a much lower need for somatic hypermutation as well as affinity maturation, leading to lower titers of antibody [92]. The larger repertoire, along with other host responses such as having constitutively expressed IFN reducing viral load, is suggested to explain why bats develop non-robust antibody responses during viral infection allowing for virus replication within the host [39,82]. 

For some hantaviral infections, the differences in immune responses between reservoir and nonreservoir host are evident in the cytotoxic T lymphocytes (CTL) response (see reviews [93,94,95]). *Seoul orthohantavirus* (SEOV) infection of animals without a functional T cell response (e.g., nude rat model) succumb rapidly to infection and diseases suggesting that cell-mediated immunity plays an important role in controlling infection [96]. Mouse models of transient and persistent infection for HTNV [97] were used to analyze the immune response of virus specific CD8+ T cells with MHC tetramers [98]. They showed that N-specific CTLs are strongly regulated and suppressed in this persistently infected mouse model by an unknown mechanism, whereas they are upregulated in the transient model [98]. Viral replication in immune cells such as monocytes, macrophages, or T cells can interfere with or actively suppress immunity and cause persistence [99,100,101]. Taruishi et al. [98] proposed that the infection of immune cells in the spleen early in infection may result in the suppression of the CTL response. In their persistent animal model experiments, the infection of the spleen correlates with changes in CTL responses. Consequently, due to the downregulation of the CTLs, some of the endothelial cells may remain infected, resulting in a persistent infection in the natural reservoir. In addition, it is hypothesized that the regulatory T cells (Treg) are activated early in the infection process, resulting in decrease in the CTL response [49,50]. Treg responses are suggested to enable a persistent infection by suppressing innate immunity, proinflammatory, and effector T cell activity in SEOV-infected, Norway rats (*Rattus norvegicus*) and SNV-infected deer mouse [43,77,101,102,103]. In these studies, Forkhead box P3 gene expression and TGF-β1–expressing b1 Treg cells were elevated during persistence, potentially interfering with viral clearance and limiting pathology [99]. 

As with hantaviruses, the outcome of arenavirus infection is mediated by CTL responses [104,105,106]. During chronic lymphocytic choriomeningitis virus (LCMV)-Cl13 infection, CTLs become functionally unresponsive or exhausted and are unable to kill virally infected cells or produce antiviral cytokines [107]. Following infection, the functional capacity of CTLs is lost in a hierarchical manner based upon the amount of available antigen [108]. When there is little virus present, CTLs maintain their functional activity, but as viral load increases, all CTL effector functions are lost, including IL-2, TNF-α, and IFN-γ production [108]. Exhausted LCMV-Cl13-specific CTLs have been shown to upregulate several inhibitory receptors including programmed death-1 (PD-1), lymphocyte activation gene-3 (LAG-3), CD39, and CTL-associated antigen 4 (CTLA-4) [109,110,111,112,113]. It has been suggested that these distinct transcriptional signatures may attenuate signaling through the T cell receptor (TCR), facilitating virus persistence [109]. Recently, the transcription factor TOX was identified as the master regulator of CTL exhaustion during chronic LCMV infection [114]. Moreover, the phenotypic landscape of virus specific CTLs was shown to differ by tissue indicating that tissues, with a higher percentage of functionally exhaustive CTLs may provide a niche for viral persistence [106,115].

For bats, less is known about the T cell responses following viral infection due to the lack of available immunological reagents; however, some groups have begun phenotypically and functionally characterizing bat lymphocyte subsets [116,117]. Gomez et al. [116] identified CTL and T helper cell populations in the black flying fox using commercially available anti-human/mouse antibodies. They observed an unusually high proportion of CTLs in the spleen, and the T cell populations within the spleen expressed IL-17A, IL-22, and TGF-β, indicating polarization toward Th17 and Treg cell subsets [116]. These findings suggests that the bat immune system may be armed and ready to thwart viral infection. However, this may not be generalizable across bat species as differences in lymphocyte populations have been reported [117]. Therefore, it would be of great interest to examine the steady state T cell responses across different wild bat species.

### 2.3. Behavioral and Demographic Characteristics

Unlike most rodents, most species of bats are social, roosting in groups which may range from a few individuals to colonies of many millions of individuals, including the largest single-species aggregations of mammals on earth except for *Homo sapiens*. Some, such as the straw-colored fruit bat (*Eidolon helvum*, Family Pteropodidae) are tree-roosters and long-distance migrators. However, the majority of massively colonial species are cave-roosters, where they congregate in maximally close proximity [118]. Examples include several species of Egyptian rousette bats, *(Rousettus* spp., Family Pteropodidae), bent-winged bats, *Miniopterus* spp. (Miniopteridae), Egyptian slit-faced bats, (*Nyteris thebaica*, Nycteridae), and several species of free-tailed bats (e.g., *Chaerephon plicatus, Mormopterus* spp., and *Tadarida brasiliensis*, Family Molossidae)) [119,120,121,122,123,124].

Bat species that live in social groups, whether in small groups or in massive colonies with close contact, must play a key role in viral maintenance in the population. In large permanent colonies, viral transmission can potentially follow the birthing cycle of pups, in which an increase in population size with naive (newborn) members reduces herd immunity, allowing for viruses to be reestablished cyclically within a colony [125,126]. Recently, individuals of the Egyptian rousette bat were found to self-isolate when immunologically challenged, a behavior which presumably would reduce pathogen contagion within the colony [127]. Notably, the bat species which have been implicated most frequently as primary reservoirs of zoonotic RNA viruses include *Pteropus* spp. (NiV, HeV, *Menangle pararubulavirus*, *Tioman pararubulavirus*) and *Rhinolophus* spp. (SARS-CoV related viruses) [128]. These bats are colonial, although not massively so.

Calisher et al. [21] list and discuss many of the viruses associated with bats, as well as several unique features of bats which may enhance their suitability as viral reservoirs. Bats’ capacity for flight (mobility, escape from predators), their long evolutionary history and broad taxonomic diversity, diverse population structure (discussed above) and social/colonial strategies, wide geographical distribution and ability to migrate (virus dispersal and spread), their distinctively high longevity, and seasonal hibernation in some temperate species (viral persistence) may all contribute to making them suitable reservoir hosts [21,69,129,130,131,132,133].

As with bats, the behavioral and demographic characteristics of rodents may contribute to their capacity to serve as reservoirs for harboring and maintaining viruses. In comparison, trade-offs between reproduction and lifespan constitute the life-history of rodents. Rodents generally exhibit an r-selected life history, characterized by early sexual maturity and large litter sizes [41], which leaves them vulnerable to resource depletion or climatic variation. Consequently, rodent populations fluctuate with seasonality and climate change, which affects the availability of food resources and, therefore, reproductive activity [134]. This, in turn, impacts the prevalence of some RNA viruses in the population. For example, in areas with large seasonal variations such as the temperate forests of Central Europe, beech mast seedings have been shown to trigger population surges of the bank vole in the following year and correlate with human PUUV outbreaks [135]. Conversely, during periods of drought, when food resources are limited, *Mastomys natalensis*, natal multimammate mice, the reservoir of LASV, are found in high abundances within human dwellings, which likely drives the higher rate of human LASV infections [136]. Even on a local scale, significant hantaviral seroprevalence differences have been observed in deer mice [137], and microhabitat preferences have been reported to vary seasonally and interannually in two sympatric reservoirs for hantaviruses in Paraguay [138]. Moreover, habitat associations of seropositive individuals differed from the general population of *for Akodon montensis* [139]. In general, for a virus to persist in a reservoir with short generation time and rapid population turnover, it must be maintained once the virus reaches a persistent and low level of replication. Calisher et al. [140] found evidence that although deer mice individuals typically lived less than a year, a few long-lived persistently infected individuals are the principal trans-seasonal reservoir of SNV.

Some viruses within the *Arenaviridae* and *Hantaviridae* persist for the lifetime of the reservoir host, with the viruses present at relatively low levels in the host [141,142,143]. However, persistent infection as a mechanism of maintenance in the reservoir population may depend on the age of infection. For arenaviruses such as *Guanarito mammarenavirus*, JUNV, LASV, LCMV, MACV, and Morogoro virus, studies have shown an age-dependency in the duration of infection among reservoir hosts [144,145,146,147,148,149,150,151]. Neonates develop long-term viral persistence, while adults can clear infection [144,145,146,147,148,149,150,151]. The mechanism by which virus persists or is cleared is an area in need of additional investigation. Experimental infection studies of captive bats have shown that some viruses (e.g., HeV and MARV) have relatively short acute infections lasting days to months, followed by the virus being cleared from the host due to the animal creating antibodies against the virus [64,65].

Regardless of whether the virus persists in its reservoir host, the maintenance of virus in the population depends on the value of the basic reproduction number, R_0_, which represents the average number of secondary infections possible from a primary infected individual [152]. When the value of R_0_ falls below 1.0 and remains below 1.0 for several generations, then the virus will eventually be eliminated from the population. We have limited information on the R_0_ of RNA viruses in bat and rodent populations and how it is affected by physiological and environmental factors as well as age, the duration of infection, and animal behavior.

### 2.4. The Inherent Genetic Plasticity of RNA Viruses

All the viruses discussed herein replicate and transcribe their genomes using an RNA dependent RNA polymerase (RdRp) encoded in their genomes. The RdRp and other viral proteins that support replication do not usually correct misincorporation of nucleosides—an exception being coronaviruses and other members of its family that can proofread mismatched base pairings during replication. Thus, replication and transcription have a high rate of error ranging from 10^−3^ to 10^−5^ per round of RNA synthesis [153,154,155]. For a 10,000 nt genome, a 10^−4^ rate of misincorporation results in, on average, one error per round of replication. As discussed by Peck and Lauring [156], the measurement of mutation rates is complex, situational and may be biased against lethal mutations, which may result in an underestimate of the mutation rate [156]. Genetic mutations arise randomly through error-prone viral replication machinery and may be selected for in the subsequent cycle of infection and replication if they confer some advantage in the reservoir. Upon infection, RNA viruses infect as quasispecies (a population of heterogenous virions) [141,153,157,158,159]. Emergence of a zoonotic virus within a naive, nonreservoir host species may follow a bottleneck where only a few genotypes survive, and even these may require additional genetic changes within the viral population for successful transmission and adaptation to the new host species (e.g., by suppressing innate immunity as discussed above) [2,6,160,161]. 

Examples of genetic mutations in viruses harbored by wildlife reservoirs that emerge and play a role in promoting reservoir-to-human and sustained human-to-human transmission are few but often involve the binding and/or entry of the virus to host cells, enhanced replicative ability in the new hosts, and/or suppression of innate immunity. Mutations enhancing reservoir-to-human transmission were reported in the first SARS-CoV spillover, where mutations in the virus’s spike protein increased the protein’s binding affinity to angiotensin converting enzyme 2 (ACE2) receptor of either the intermediary host, Asian palm civets (*Paradoxurus hermaphroditus*) or humans [162]. Mutations occurring after initial spillover of EBOV from its reservoir host into humans were observed in the 2013 West Africa outbreak, where a mutation in the virus’s host cell-binding glycoprotein resulted in increased infectivity [163]. The mutation was primate specific, and ultimately, the mutation was associated with an increase in human mortality. 

## 3. A Look at Environmental Factors That Drive Spillover of Viruses in Bat and Rodent Populations

From the first recognition of virus spillover from nature, scientists have endeavored to understand the drivers behind the emergence of new viruses. Factors such as climate change, land use change (i.e., logging, burning, agriculture), human expansion into habitat (i.e., recreation, farming), and land fragmentation (i.e., roads, buildings, towns) are some of the key drivers which seemingly alter wildlife populations and hence the balance of the virus in reservoir populations and drive spillover [2,164,165,166]. Each of these extrinsic pressures may impact the animal population by alteration of habitat (e.g., size, composition, fragmentation), resource availability (e.g., water, food, refuge), and/or community structure (e.g., species richness and diversity, population density and demographics) [2,139,167,168,169]. Any one of these, or a combination thereof, could influence host well-being and contact rates and thus may drive spillover of infectious agents into new host species. Complicating matters further, habitat preferences of abundant rodent species may be seasonally and interannually variable, resulting in probable temporal variation in interaction rates among reservoir and nonreservoir species [170]. Similarly, human activities may vary seasonally, altering their exposure and risk. Additionally, human cases may result from seasonal increases in rodent population densities and the movement of rodents into homes and barns during the colder seasons [2,171].

The ecological drivers of outbreaks by emerging and re-emerging zoonotic viruses are not well understood due to the challenges in designing controlled field studies to understand how each of these specific factors drive pathogen presence and prevalence in wild animal populations, and during an outbreak, surveillance is usually not conducted with this in mind. For example, during the outbreak of hantavirus pulmonary syndrome (HPS) in the southwestern USA in 1993 researchers surveyed animals in the vicinity of the trailers and homes of infected persons. This resulted in the identification of the deer mouse, as the reservoir of the causative agent, SNV. In nature, the prevalence of hantaviruses in rodent populations can vary from undetectable to 40% in the primary rodent reservoir [137,140,168,172,173,174]. A persistent conundrum of rodent-borne virus outbreaks that occur over large areas (e.g., southwestern USA, southern Argentina) is that cricetid and murid rodents live in relatively small habitats and conduct limited travel during their life span (usually < two km^2^) [175]. This raises the question of whether all the mice in a particular outbreak zone respond similarly to an ecological or environmental change in situ or if viruses carried by the reservoir move across rodent populations as a traveling wave as one model suggests [176]. Retrospective analyses on the environmental characteristics of sites where people were infected with those at sites where people were not infected suggested that the areas impacted by El Niño-Southern Oscillation (ENSO) had a greater risk [177]. Nearly 20 years later, we have no concrete evidence, other than this, as to what drove the outbreak in the desert southwest. A second strong ENSO occurred in 1997–1998, and HPS rose 5-fold in the southwestern USA suggesting it as a clear factor [178].

It has been suggested that the most important drivers of spillover of bat-borne RNA viruses to humans is the anthropogenic encroachment into natural habitats of bats through urbanization and agriculture [125,179,180]. For example, anthropogenic deforestation as well as climate anomalies are proposed to have played a role in the outbreak of NiV. Large forest areas were burnt producing extensive haze, which drove the bats to follow an irregular migration. The area was also undergoing a severe drought caused by an ENSO event. These two factors combined drove bats carrying the virus to move into areas where pig barns were located, thereby transmitting NiV to pigs [181]. Outbreaks of filoviruses and henipaviruses correlate to birthing pulses of pups in the bat population [64,125,182]. 

Clearly, reservoir population density and the prevalence of the virus in the population contribute to the probability of increased prevalence of the virus in its reservoir and the probability of spillover. In general; however, very little is known for many viruses in terms of how these viruses are maintained within their natural reservoirs, the normal prevalence of virus in wildlife, genetic variation within host and communities, and what drives increased spread in reservoir populations. In addition to natural history study of reservoir-virus populations in their native habitats, experimental field studies would help identify factors that change virus–host population dynamics.

## 4. How Do Viruses Spillover from Bats or Rodents to Humans?

**Figure 1:**
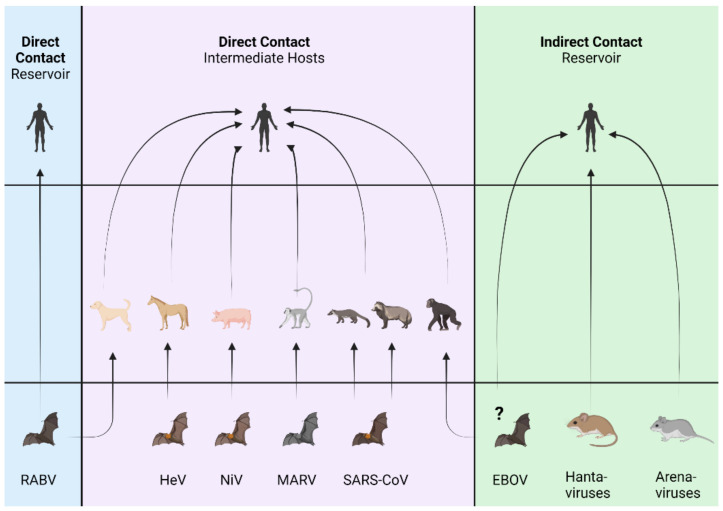
Major routes of spillover transmission of viruses harbored by bats and rodents. Three of the most common routes of exposure from bats or rodents to humans are depicted. As illustrated, more than one route may occur. Abbreviations: RABV, rabies viruses; HeV, *Hendra henipavirus*; NiV, *Nipah hendravirus*; MARV, *Marburg marburgvirus*; SARS-CoV, severe acute respiratory syndrome coronavirus; EBOV, *Ebolavirus* species.

The majority of spillover events from a wild rodent (Supplementary Table S1) or bat (Supplementary Table S2) species that results in infections of humans begin with direct or indirect contact (Figure 1) [7]. Direct contact between humans and rodent or bat reservoir hosts may occur through interactions such as touching animals shedding virus, activities associated with hunting and harvesting bushmeat, human consumption of bushmeat or contaminated food, or being bitten. Indirect contact with a virus from a rodent reservoir to a human mainly occurs through inhalation of aerosolized excreta from infected rodents [2]. Exposures to excreta have been associated with human activities such farming, camping, or outdoor military exercises in areas that are contaminated with the reservoir excreta. Similarly, inhalation of bat guano has been suggested as a potential route of exposure as suggested by human cases of Pteropine orthoreoviruses, coronaviruses, filoviruses and others that have occurred following visits to workplaces or caves where bats roost [183,184,185]. Many exposures are associated with housing or other buildings which rodents have invaded and occupied or have left excreta [186,187,188,189,190,191]. Direct or indirect contact may also occur via interactions with an intermediate spillover host (Figure 1). 

In the following, we briefly review the above scenarios of spillover that resulted in human cases of disease and characteristics of these events. 

### 4.1. Spillover Associated with Direct Animal Reservoir Contact 

*Rabies lyssavirus*, genus *Lyssavirus*, was the first bat-borne virus discovered, which in retrospect is unsurprising because 50% of the currently known viruses carried by bats are rhabdoviruses [19,192]. The first reported outbreak of rabies happened in 1929 in Trinidad when 17 fatal human cases were reported along with a similar disease in nearby cattle [32]. Dr. Pawan, the island’s bacteriologist, found that rabies virus was transmitted from the bite of vampire bats to humans and cattle [32]. In the subsequent decade, Pawan further showed that insectivorous and frugivorous bats can harbor the virus as well [192]. Later in the United States, in addition to insectivorous bats, raccoons, skunks, and foxes were identified as additional reservoirs of rabies virus variants [193]. According to the WHO, there are approximately 59,000 human deaths from rabies in over 150 countries each year. Currently, 99% of all rabies cases comes from one intermediate host, dogs, through bites. In the US, rabies in dogs has been eradicated, and hence while the cases are low, rabies cases originate in bats, coyotes, raccoons, and skunks [194]. In addition to bites from rabies-infected animals, other animal bites are also associated with human cases of disease for several additional viruses [195,196,197]. 

Human cases of infection have been associated with consumption of their reservoirs for both bats and rodents. In certain areas, eating rodent bushmeat is considered a delicacy; therefore, human transmission can also occur by exposure to their bodily fluids (e.g., viremia in blood [64,197]) during hunting and food preparation [198,199]. Hence, it is not clear whether the consumption or the hunting was the major risk factor. Several cases of LASV have been associated with the capture and consumption of rodents [199]. 

### 4.2. Spillover Associated with Direct Contact: Intermediate Hosts

Some outbreaks in humans involving paramyxoviruses, coronaviruses, and filoviruses have occurred through contact with an intermediate host that acts as an amplifier host [179,200,201,202,203]. For example, palm civets and raccoon dogs (*Nyctereutes procyonoides*) from live markets may have played a crucial role in outbreaks of human diseases such as those caused by SARS-CoV [202,204]. Since coronaviruses in nature were very poorly sampled until the 2002–2003 SARS outbreak, there is no data that definitively shows whether direct or indirect transmission is more plausible [205]. The bat reservoir for SARS-CoV-2 has yet to be identified and an intermediate host has yet to be confirmed. In both instances, it has been suggested that a spillover occurred from a bat to an animal sold for human consumption in a live wildlife market [14,202]. In the case of MERS-CoV, research supports that spillover occurred from bats to dromedary camels, and camels transmit the virus to humans presumably through contact with nasal discharge [203,206,207,208]. 

MARV emerged in 1967 in Marburg and Frankfurt, Germany, as well as in Belgrade, Serbia, when 31 cases were reported of an unknown disease to which seven patients succumbed [209]. Epidemiological evaluation of the three outbreaks reported that those infected were working with vervet monkeys (*Chlorocebus pygerythrus*) that were imported from Uganda [210,211]. The search for the reservoir of MARV was a lengthy process, and in 2009 the putative reservoir was identified through molecular detection methods as the Egyptian rousette bat [212,213,214]. 

In September 1976, *Zaire ebolavirus* (ZEBOV) emerged in Zaire (now the Democratic Republic of Congo) and resulted in 318 cases with a mortality rate of 88% [215]. The first case occurred in a village near the Ebola River in Zaire. The man was an instructor at the Mission School, who, while traveling, purchased fresh and smoked antelope and monkey meat but only ate the antelope [215]. Outbreaks of EBOV occurred throughout Central and Western Africa in 1994 and 2000, and the largest outbreak occurred during 2013–2016 with sporadic outbreaks continuing. Extensive searches have been conducted to identify the reservoir, but it remains elusive [216,217]. Bats have been proposed to harbor the viruses but have not been proven to be the reservoir [34,37]. While the reservoir for the virus has yet to be identified, numerous outbreaks have occurred following humans handling animals such as primates (chimpanzees and gorillas) and antelopes (duikers) and their carcasses [218,219,220]. The presence of EBOV in a number of human bodily fluids enables very efficient person-to-person transmission [221].

There are two species of the *Henipavirus* associated with outbreaks of human disease, HeV and NiV; these viruses are carried by multiple species of the bats commonly referred to as flying foxes (*Pteropus* spp.) [222,223,224,225]. HeV first emerged in Brisbane, Australia in 1994 when two human and 21 horse cases of an unidentified respiratory disease were reported, resulting in one human fatality and 14 horses being euthanized [200,201]. Since the initial outbreak, cases have been reported in Australia from interactions of humans with horses infected with the virus [226,227,228]. Although transmission of HeV from flying foxes to horses has yet to be demonstrated, it is hypothesized that transmission occurs when horses encounter urine from infected bats [229,230]. 

The first outbreak of NiV occurred in Malaysia during 1998 in pigs and humans, with 265 human cases (105 fatal) and the culling of nearly one million pigs [231,232]. The earlier outbreak of HeV suggested bats as a reservoir, and ultimately, seroprevalence surveys of bats showed that multiple species of bats had neutralizing antibodies to NiV, and the virus was isolated from the urine collected from island flying foxes [233,234,235]. Spillover of NiV to pigs is thought to occur when pigs consume fruits that have been partially eaten by bats or via exposure to the excreta of infected bats [180]. In addition to contact with pigs, bat-to-human spillover is thought to occur through the consumption of date palm sap that has been contaminated with NiV from bat excreta [236]. Outbreaks of NiV with 40–70% fatality have since been reported in Bangladesh, India, and the Philippines [237,238,239]. Significantly, in Bangladesh and India, epidemiological evidence also indicates human-to-human transmission through close contact.

### 4.3. Spillover Associated with Indirect Animal Reservoir Contact 

Epidemiological evidence suggests the indirect mode of transmission (aerosolized excreta) from rodents or bats to humans is similar for many zoonotic viruses. The literature supports indirect contact via inhalation of rodent excrement or direct contact with rodents such as rodent bites (discussed above) as the main routes of transmission to humans [2]. These exposures occur during certain types of human activities that are often in rural areas or in the natural environment or habitat of the reservoir. We will highlight seminal examples of specific human activities associated with outbreaks in human populations.

#### 4.3.1. The Role of Agriculture

In South America, human cases of HPS, Argentine hemorrhagic fever (AHF), and Bolivian hemorrhagic fever (BHF) have been associated with agricultural activities. In the following we briefly discuss two notable outbreaks of arenaviruses associated with agricultural activity. Seven species of arenaviruses are known to cause hemorrhagic fever in humans in South America and Africa, presumably through the inhalation of aerosolized infectious excreta [240,241,242]. 

After its isolation in 1959, JUNV was the second arenavirus (following LCMV in 1933 in the USA) recognized to cause human illness, AHF [243,244]. The rodent reservoirs of JUNV, *Calomys musculinus* (dry lands vesper mouse) and *C. laucha* (small vesper mouse), are endemic to the Humid Pampas, a farming region in central east Argentina [245]. *C. musculinus* is believed to be the primary reservoir of JUNV, although antibody to the virus is also detected in *C. laucha* [245]. Rodent population density is positively correlated with JUNV seroprevalence. From these field studies of the reservoir, the main route of transmission is likely horizontal (fewer infections of juveniles and subadults and there is higher prevalence among males). The emergence of AHF is hypothesized to have occurred due to intensive deforestation and agriculture practices that promoted contact between humans and the rodent reservoirs. Since the first recognition of AHF in 1958, annual epidemics have been reported ranging from several hundred to 3500 cases, with most cases occurring between April and July (late fall and early winter) [244,246]. These outbreaks coincide with the corn harvesting season, which is thought to cause an increase in rodent population densities and to promote human-rodent contact. In fact, AHF mainly affects male rural workers. 

Around the same time as JUNV was recognized, human outbreaks of MACV associated with Bolivian hemorrhagic fever (BHF) were reported in Bolivia [247]. The reservoir host of MACV is *C. callous* [248]. Outbreaks of BHF emerged in Beni Department, Bolivia during 1959, following political instability that led families living in rural areas to rapidly transition to subsistence agriculture [247]. It was reported that an increase in the use of DDT on crops for insect control coupled with abnormally low rainfall led to a decline in cats, resulting in an increase in rodent numbers. After 1959, there were multiple outbreaks in rural communities in Bolivia, which continued until 1964 with the implementation of rodent control measures (mouse traps and poisoning with zinc phosphide) [249]. Rodent control programs developed in San Joaquín during the outbreak of BHF in 1962 killed ca. 3000 *C. callosus* in a span of 4 months, demonstrating that this rodent can acquire high population densities around human habitations. During 18 months of research in Beni, Bolivia cases of BHF were reported in every month of the year, with a distinct increase from January to May a period of intense agricultural activity (rainy season) [247]. It was not until 1963 that MACV was isolated from the spleen of a patient who succumbed to disease [54]. Since its emergence, there have been sporadic outbreaks of BHF which have been related to agricultural occupational exposure. Cases are highest between April and July, which are the late rainy and early dry seasons, as *C. callosus* invades homes during the rainy seasons. However, only a handful of cases have occurred since the 1960′s. In 2007–2008, an outbreak of five cases was reported among farmers in Bolivia [250].

#### 4.3.2. The Role of Caves, Rural Workplaces, and Homes

After the first cases of MARV through direct contact with nonhuman primates, several other large outbreaks occurred throughout central and southern Africa; however, in contrast to the interaction with an intermediate nonhuman primate host discussed above, these cases were associated with patients visiting caves [251,252,253]. 

The first human cases of *Sudan ebolavirus* (SUDV) likely originated in a cotton factory in Nudar, Sudan in June 1976. In the five months that followed, 284 cases were reported with a mortality rate of 53%. It is suggested that exposure occurred via excreta from bats roosting in this factory [254].

Cases of Lassa fever were recognized in the 1950s; however, the virus causing the illness, LASV, was not identified until 1969 when three nurses died from it in a mission hospital in Nigeria [255,256]. Many of the early outbreaks were localized within hospital wards, where nurses and doctors became exposed to patient bodily fluids [257,258,259]. Now, nosocomial infections are uncommon, and human transmission occurs mainly in village homes through direct contact with rodents or indirectly by consumption of contaminated food products, exposure to surfaces contaminated with rodent excreta, or inhalation of aerosolized virus [260]. Additionally, several cases have been recognized to be imported from individuals traveling from West Africa [261,262]. In West African villages where LASV is endemic, homes are usually devoid of food storage spaces such as cabinets (cupboards), so food is stored in plastic buckets or large flour bags on the ground or hung from ceilings or walls [263]. During the dry season (January–March) when there are limited food resources, *M. natalensis* tends to aggregate in homes in search for food [136]. These rodents excrete virus in their urine; therefore, an infected rodent can indirectly transmit the virus by urinating on food products [150]. More recently, the African wood mouse, *Hylomyscus pamfi*, in Nigeria and Guinea multimammate mouse, *Mastomys erythroleucus*, in both Nigeria and Guinea were recognized as additional hosts [264].

In the first outbreak of SNV in the summer of 1993 through 1995 in the Four Corners states of the southwestern USA, 69% of the hantavirus pulmonary syndrome (HPS) cases had exposures closely associated with peridomestic activities in homes that showed signs of rodent infestation [191]. A retrospective analysis of the outbreak of SNV during 1998–1999 noted that most HPS case patients reported indoor exposure to deer mice [178]. In the fall of 2012, a cluster of cases occurred in Yosemite in deer mice infested tent cabins in Curry Village [265]. Similar observations were made in outbreaks in Paraguay, while in Argentina most cases over a 13-year period were associated with agriculture [266], although there are reports suggesting peridomestic exposure [267].

#### 4.3.3. The Role of War

The first recorded outbreak of hemorrhagic fever with renal syndrome (HFRS) associated with the hantavirus, HTNV occurred in soldiers during the Korean War in the early 1950′s and probably resulted from outdoor activities such as sleeping in tents and performing military exercises [268]. Similarly, cases of HFRS occurred in soldiers during the war in Bosnia [269] and in military training exercises of USA soldiers in Germany [270]. As noted in ecological surveillances of military sites in Korea [271,272], reservoir mice harboring hantaviruses are common in training areas with mortar ranges and troop maneuver/ assembly areas and pose a threat for exposure. 

#### 4.3.4. Person to Person Transmission following Spillover

Most human infections with hantaviruses and arenaviruses are associated with indirect exposure of rodent excreta. However, viruses in each genus have had suspected or documented cases of person-to-person transmission. In one report of illness from MACV, a 20-year-old female nursing student living in Cochabamba, Bolivia became ill following travel to her hometown in Fortaleza in Beni Department in December 1970 [273]. Upon return to Cochabamba, she was hospitalized and died January 28. Subsequently, five individuals who had direct contact with her in the hospital became ill and all died, including her father, nurses, and the pathologist who conducted her autopsy. More recently in 2008, a case of hemorrhagic fever caused by *Lujo mammarenavirus* involved a 36-year-old female travel agent who worked on a peri-urban farm near Lusaka, Zambia. Following illness, she was flown to Johannesburg, South Africa to be treated at a private hospital. There, four healthcare workers were exposed and became ill and three died [274]. Among the hantaviruses, there have been documented cases of person-to-person transmission only for the South American *Andes orthohantavirus* (ANDV) which have occurred in Argentina and Chile [267,275,276,277,278,279]. In 1996, the first human-to-human outbreak of ANDV was reported in southwest Argentina among 18 people [267]. The outbreak began on 22 September 1996 and lasted until 5 December 1996. The index case was a 41-year-old male who lived in the rural town El Bolsón. Twenty days after he became ill, his doctor became ill, followed by his 70-year-old mother a day later. Subsequently, several cases were reported from small household clusters, nosocomial infections, and from a car trip to the funeral of the 70-year-old mother. No evidence of nosocomial infections has been reported in Chile where ANDV is endemic [280]. However, transmission occurs mainly through household contacts and risk is especially high among sexual partners [278].

## 5. Conclusions

The number of novel or reemerging RNA viruses which cause infections in humans increases each year. While most of these zoonotic infections do not lead to a pandemic, the global public health burden of all combined infections is high. The 2002–2003 SARS-CoV epidemic resulted in more than $40 billion in losses despite causing fewer than 1000 deaths [281]. Predicting the next outbreak, epidemic or pandemic caused by an RNA virus will remain an on-going challenge for the foreseeable future. Predictive models will require a deep understanding of the intrinsic and extrinsic drivers of virus spillover in their native habitat and of how these viruses are maintained in their native reservoir. Whether a virus can persist for the lifetime of a reservoir host or can be cleared, the mechanisms that drive these outcomes is an area in need of expanded investigation, as there is a lack of literature for many reservoir species and viruses in this regard. Experimental animal reservoir models are essential for understanding these host–virus dynamics, and even conservative efforts to expand the taxonomic diversity of experimental models would prove worthwhile. Alternative rodent and bat experimental models of infection have provided important information as highlighted herein. In addition to expanding reservoir models, well-designed, targeted, empirical surveillance studies that incorporate immunological and pathological examinations may provide crucial information about the effect of natural infections. The principal challenges of field studies are largely technical; for example, identification of specific stages of infection of viruses in animals in nature while in the field in is unlikely, and the ability to isolate or detect virus in a persistent infection in the laboratory may be difficult if below the limit of detection, requiring more samples than in standard, controlled laboratory studies. Metagenomic approaches have improved in recent years and may eventually overcome technical limitations of detecting virus in field-sampled, persistently infected reservoirs. However, integration and interpretation of this information with immune response and pathology specific to the virus of interest will be challenging, as many animals collected in the field harbor other pathogens. Hence, this underscores the need to establish laboratory colonies of the natural reservoirs to benefit understanding. The difficulties of establishing alternative, reservoir models are numerous and challenging, but important for advancement of understanding biological mechanisms. 

In addition to the natural reservoirs, intermediate spillover hosts (e.g., agricultural livestock and wildlife species that are kept as pets or taken for food consumption) play an important role in the transmission network of viruses to humans. As such, intermediate hosts acting as a bridge between natural reservoirs and humans warrants greater attention for advancing our understanding of virus transmission and maintenance from these hosts. In addition to surveillance of bats, efforts need to focus on intermediate hosts as many bat viruses’ transmission to humans relies on these amplifier hosts. Metagenomic surveillance of potential intermediate hosts would provide valuable information on circulating and newly introduced viruses. Maintaining such samples in tissue biorepositories for wildlife specimens in museums is critical for retrospective analyses. 

In areas where reservoirs and intermediate hosts are known, control measures should be used to keep these reservoirs in check, such as preventing bats and rodents from contaminating food sources. Controlling the further spread of outbreaks is an important aspect of study to prevent isolated outbreaks from progressing into epidemics or pandemics. One essential feature to controlling outbreaks is understanding transmission routes to limit further spread, e.g., aerosol with SARS-CoV-2 and exposure to infected bodily fluids in the case of EBOV. Controlling virus transmission during outbreak scenarios depends on virus population dynamics within the reservoir and intermediate hosts. Understanding virus populations and their variants within reservoir and intermediate hosts are important as inherent mutations and mutations acquired through transmission may lead to an increase in viral fitness, allowing for spillover and increased transmission, as seen in the case of SARS-CoV and the Asian palm civet. Additionally, host genetics needs to be investigated as host genetic background can influence infection outcome. Some host population members can have a higher likelihood of transmitting a virus compared to others within the same population (e.g., superspreaders). Efforts focused on virus surveillance, virus–host interaction, and virus population changes can be complicated as reservoirs, intermediate hosts, and the viruses they harbor lack necessary reagents for their study, so development of these reagents are essential. In summary, we have addressed (1) why zoonotic viruses reside in bat and rodent reservoirs, (2) environmental and ecological causes driving spillover, and (3) shared and unique characteristics of previous epizootic events. Further investigation of these topics is a long-term process that will take vigorous effort, but continuous investigation will provide an effective means to understand the relationship between spillover and emergence of bat- and rodent-borne RNA viruses in their hosts.

## Supplementary Materials

The following are available online at https://www.mdpi.com/article/10.3390/v13081509/s1, Table S1: Examples of Outbreaks in Human Populations by RNA Viruses Harbored by Rodents, Table S2: Examples of Outbreaks in Human Populations by RNA Viruses Harbored by Bats. References [282,283,284,285,286,287,288,289,290,291,292,293,294,295,296,297,298,299] are cited in the Supplementary Materials.
