# Supplementary material for: Common Themes in Zoonotic Spillover and Disease Emergence: Lessons Learned from Bat- and Rodent-Borne RNA Viruses
Source: Viruses. 2021 Jul 31;13(8):1509. doi: 10.3390/v13081509 (PMC8402684; doi:10.3390/v13081509)
Supplement: Supplementary file 1 [file viruses-13-01509-s001.zip › viruses-1202993-supplementary.pdf]

SupplementaryTable S1. Examples of Outbreaks in Human Populations by RNA Viruses Harbored by Rodents

**Virus Common Name**	**Virus Species Name (ICTV)**	**Family**	**Notable outbreaks**	**Reservoir**	**Citations**
Chapare Virus	*Chapare mammarenavirus*	*Arenaviridae*	2003-2004; 2019 Bolivia	Unknown	[286,287]
Guanarito Virus	*Guanarito mammarenavirus*	*Arenaviridae*	1989 Venezuela	*Zygodontomys brevicauda*	[288,289]
Hantavirus	*Hantaan orthohantavirus* *Sin Nombre orthohantavirus*	*Hantaviridae*	1950-1953 Korea 1993 Four Corners, USA	Various rodent spp.	[290-292]
Junin Virus	*Argentinian mammarenavirus*	*Arenaviridae*	Recognized ca. 1950 Argentina	*Calomys musculinius*	[243,246]
Lassa Virus	*Lassa mammarenavirus*	*Arenaviridae*	Recognized ca. 1950 West Africa	*Mastomys natalensis*	[255,293]
Lujo Virus	*Lujo mammarenavirus*	*Arenaviridae*	2008 Zambia and South Africa	Unknown	[294]
Lymphocytic Choriomeningitis Virus	*Lymphocytic choriomeningitis mammarenavirus*	*Arenaviridae*	Recognized ca. 1933U.S.A.	*Mus musculus*	[295,296]
Machupo Virus	*Machupo mammarenavirus*	*Arenaviridae*	1950s-60s Bolivia	*Calomys callosus*	[297]
Sabiá Virus	*Brazilian mammarenavirus*	*Arenaviridae*	1990 Brazil	Unknown	[298,299]

Supplementary Table S2. Examples of Outbreaks in Human Populations by RNA Viruses Harbored by Bats

Virus Common Name	Virus Species Name (ICTV)	Family	Notable outbreaks	Reservoir	Citations
Ebola Virus	*Sudan ebolavirus* *Zaire ebolavirus*	*Filoviridae*	1967, 2014-2016 West Africa	Fruit bats (proposed)	[34,37,215]
Hendra Virus	*Hendra henipavirus*	*Paramyxoviridae*	1994 - present Australia	*Pteropus* spp.	[200,201,222,223]
Marburg Virus	*Marburg marburgvirus*	*Filoviridae*	1967 Germany, Serbia	*Rousettus aegyptiacus*	[209,213]
MERS-CoV	*Middle East respiratory syndrome-related coronavirus*	*Coronaviridae*	2012 Middle East, 2015 Republic of Korea	Various bat species	[203,206,282-287]
Nipah Virus	*Nipah henipavirus*	*Paramyxoviridae*	1998-1999, Southeast Asia, Sporadic in SE Asia	*Pteropus* spp.	[231,232,234]
Rabies Virus	*Rabies lyssavirus*	*Rhabdoviridae*	Recognized since 1930's Africa, Asia and Americas	Various bat species	[32,192]
SARS-CoV	*Severe acute respiratory syndrome-related coronavirus*	*Coronaviridae*	2003-2004 ~24 countries	Various bat species	[24]
SARS-CoV-2	*Severe acute respiratory syndrome coronavirus 2*	*Coronaviridae*	2019 Global pandemic	Bats (proposed)	[14]
